# Sexual Dimorphism in Immune Development and in Response to Nutritional Intervention in Neonatal Piglets

**DOI:** 10.3389/fimmu.2019.02705

**Published:** 2019-12-09

**Authors:** Zoe Christoforidou, Marina Mora Ortiz, Carlos Poveda, Munawar Abbas, Gemma Walton, Michael Bailey, Marie C. Lewis

**Affiliations:** ^1^Infection and Immunity, School of Veterinary Science, University of Bristol, Bristol, United Kingdom; ^2^Food and Nutritional Sciences, School of Chemistry, Food and Pharmacy, University of Reading, Reading, United Kingdom

**Keywords:** immune development, neonate, weaning, nutritional intervention, mucosal immunology, inulin, prebiotic, sexual dimorphism

## Abstract

Although sex disparity in immunological function and susceptibility to various inflammatory and infectious disease is recognized in adults, far less is known about the situation in young infants during immune development. We have used an outbred piglet model to explore potential early sex disparity underlying both mucosal immune development and systemic responses to novel antigen. Despite similarities in intestinal barrier function and therefore, presumably, antigen exposure, females had less CD172^+^ (Sirp-α) antigen presenting cells and expression of MHCIIDR at 28 days old compared to males, along with greater regulatory T-cell numbers. This suggests that, during infancy, females may have greater potential for local immune regulation than their male counterparts. However, females also presented with significantly greater systemic antibody responses to injected ovalbumin and dietary soya. Females also synthesized significantly more IgA in mesenteric lymph nodes, whereas males synthesized more in caecal mucosa, suggesting that plasma cells were retained within the MLN in females, but increased numbers of plasma cells circulated through to the mucosal tissue in males. Significant effects of inulin and *Bifidobacterium lactis* NCC2818 on the developing immune system were also sex-dependent. Our results may start to explain inconsistencies in outcomes of trials of functional foods in infants, as distinction between males and females is seldom made. Since later functionality of the immune system is highly dependent on appropriate development during infancy, stratifying nutritional interventions by sex may present a novel means of optimizing treatments and preventative strategies to reduce the risk of the development of immunological disorders in later life.

## Introduction

Sex plays a role in innate, humoral, and cell-mediated immunity. It is well-established that adult males are more susceptible to infectious disease (1) and generate less reactive immune profiles (2, 3), whereas adult females are more susceptible to inflammation and autoimmunity (4, 5) but fare better when it comes to infectious disease (6). Females are reported to generate significantly higher levels of protective antibodies and cell-mediated immunity following viral vaccinations against measles, rubella, rabies, smallpox, dengue, mumps, influenza, yellow fever, and hepatitis A and B (7–9). However, there are clearly complexities as other studies have reported that the incidence of immune-associated conditions such as asthma (10) and type 1 diabetes (11) is considerably higher in males than in females. These differences are likely to be attributable, at least in part, to sex steroids which can bind to immune-associated cells and impact on their function (2), but also to non-endocrine-related genes since significant numbers of immune-associated genes are located on the X chromosome (12). In addition, there is growing evidence for sexual dimorphism of intestinal microbiota in humans and other species (13–16), and this may contribute to divergent development of host immune systems (17–22). However, it is not yet clear whether this has a genetic basis, or if other determinant factors are the key drivers.

The studies regarding sex differences in immunity in young adults of all species, including humans and pigs, have tended to address the differences which may be present after sexual maturity (23). However, there is a growing body of evidence that immune differences are present very early on, even during the prenatal stages. For example, preterm females exhibit a less severe disease course and improved prognosis in many pathological states compared to male counterparts and this has been linked, in part, to differences in early immune development (24). Neonatal screening programs for detection of primary immune deficiencies show that in males, cord blood contains lower numbers of CD4^+^ T-lymphocytes, lower CD4/CD8 T-lymphocyte ratios, and higher CD8^+^ T-lymphocyte and NK cell counts than cord blood from females (25). In addition, neonatal immune challenges with LPS have been shown to induce long-term effects on cardiac development and heart function which are sex-dependent in rats. Here, male rats exhibited decreased left ventricle to body weight ratios compared to females and this was linked to delays in post-ischemic recovery following exposure to LPS (26). Differences between sexes in humeral immune responses to vaccines are variable but, where differences are observed, responses are higher in girls than in boys (27). Taken together, these data suggest that sexual dimorphic immunity is not restricted to puberty or to post-pubertal adults. However, there is a paucity of knowledge of sexual dimorphic prepubertal immunity, despite the clear implications for the treatment of infant disease.

Probiotics are “live microorganisms that, when administered in adequate amounts, confer a health benefit on the host” (28). Human-derived *Bifidobacterium lactis* NCC2818 strain has been demonstrated as having probiotic activity in humans and in rodent models. These include reduced allergy symptoms and pathogen load, and prevention or reduction of antibiotic-associated, and rotavirus-associated diarrhea (29–32). The effects of probiotics, including *B. lactis* NCC2818, during the neonatal period, a time when the resident microbiota is changing rapidly, remain unclear. However, fewer and shorter episodes of diarrhea and fewer antibiotic prescriptions have been reported in human infants receiving *B. lactis* supplemented formula milk compared to unsupplemented formula (33). On the other hand, prebiotics are “selectively fermented ingredients that result in specific changes in the composition and/or activity of the gastrointestinal microbiota, thus conferring benefit(s) upon host health” (34), largely by driving proliferation of target bacterial species such as *Bifidobacterium* and *Lactobacillus*. The prebiotic chicory-derived inulin has been demonstrated to protect against chronic inflammatory diseases by dampening immune responses through short-chain fatty acid production (35), improved intestinal barrier function and by reducing innate immune cell infiltration (neutrophils and macrophages) into the colonic mucosa (36). Inulin appears especially useful in increasing Bifidobacteria numbers in the elderly, in whom such populations are often depleted (37). However, the impact of inulin supplementation on the immune system of healthy neonates remains unclear. To the best of our knowledge, the effects of dietary supplementation with either probiotics, or prebiotics on the development of the immune system has yet to be explored in a sex specific manner. Failing to consider sexual dimorphism in immune development, and on the response to dietary supplementation in infants could, in part, explain inconsistency in results from intervention trials in both humans and other species.

Precocial piglets are important models for studies of the impact of early nutrition and nutritional supplementation on immune development since their self-sufficiency permits early separation from their mothers, thus limiting the maternal influence at this critical period of developmental plasticity. Additionally, pigs are valuable, tractable, preclinical models for humans (38) since they share many features of gastrointestinal physiology, immunology, microbiology, diet and pathologies (39–43) and are more outbred than rodent models so better reflect the human population. Given the evidence for sex-based immunological differences during early-life, it follows that nutritional immune modifiers may drive differences in the development of the immune system in a sex-specific manner. We thus hypothesize that *B. lactis* NCC2818, and inulin, will have sexually dimorphic effects on both the development of porcine mucosal immunity, and systemic antibody responses to challenge.

## Methods

### Animal Model

Animal housing and experimental procedures were all performed at the University of Bristol Veterinary Science School in accordance with local ethical guidelines. All experiments were reviewed and approved by the Bristol Animal Welfare and Ethical Review Body (AWERB) and were performed under a UK Home Office License.

**Figure 1:**
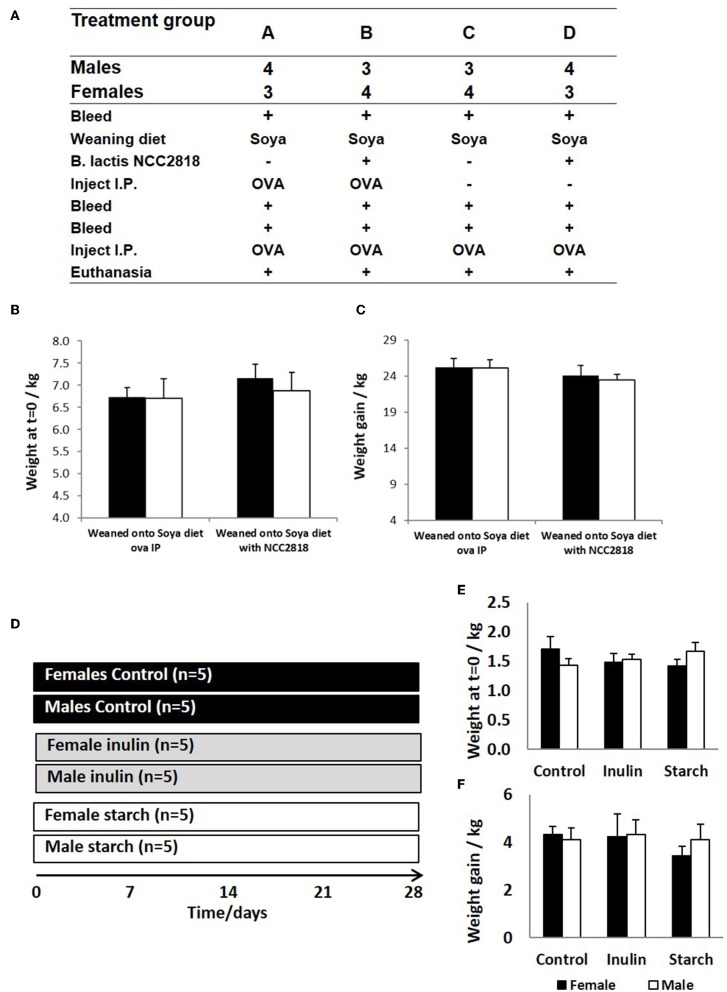
Animal trial **(A)** where 28 piglets were weaned at 3 weeks old onto diets where soya was the sole protein source (groups A–D). Half (groups B,D) were supplemented with *Bifidobacteria lactis* NCC1828. Groups (A,B) received an intraperitoneal (IP) injection of ovalbumin (OVA) with Quil-A adjuvant at 3 (primary) and 9 (secondary) weeks, while groups (C,D) received OVA IP injection with Quil-A adjuvant only at 9 weeks and this was the primary exposure. Males and female piglets were evenly distributed between the groups (*n* = 7). Weight at 3 weeks **(B)** and weight gain between 3 and 11 weeks **(C)** are presented by sex (females = black bars and males = white bars) and treatment group (group and show no significant difference between the sexes or in response to treatment. A second animal trial **(D)** where 30 piglets were removed from maternal nutrition and received bovine-based formula milk from 1 day old until the conclusion of the trial at 28 days. The formula received by 5 males and 5 females was supplemented with inulin (gray panel) whilst a further 5 males and 5 females were supplemented with starch (white panel). A further 5 males and 5 females received no supplementation (controls, black panel). Day 1 weights **(E)** and weight gain at 28 days **(F)** are presented by sex and treatment group (control, inulin, or starch). Females are denoted in black and males in white. Error bars = SEM.

In experiment 1, sex-specific effects on systemic antibody response to novel exposure to injected OVA and fed soya protein at weaning were examined. Seven outbred Large white × Landrace F1 hybrid sows were artificially inseminated using semen from a single UK standard commercial fast-grow Hylean boar (supplied by Hermitage-Seaborough Ltd, North Tawton, Devon, UK). Sows were transported to the department of Clinical Veterinary Science at the University of Bristol 6 weeks prior to parturition and fed on a wheat-based diet (BOCM Pauls Ltd., Wherstead, UK). Colostrum uptake was not assessed in any experiment. At 3 weeks of age, the resulting piglets (*n* = 28) were weaned into 4 groups (*n* = 7 per group) (Figure 1A), with sexes and litters stratified within treatment groups (2 pens/treatment) and housed on straw in standard temperature controlled large animal facilities. At this point, piglets were weaned onto a soya-based diet (Feed composition information available in Table 1) supplemented with appropriate levels of vitamins and minerals, designed to meet the nutritional requirements of pigs of this age and manufactured to order by Volac (Parnutt Foods Ltd., Sleaford, Lincolnshire, UK). The weaning diet contained 21% protein which was exclusively from soya. Half the pigs (group B and D, males and females) also received *Bifidobacterium animalis* subsp. *lactis* (CNCM I-3446, NCC2818 supplied by Nestle Ltd) probiotic diet supplementation in the form of spray-dried culture mixed into mash feed at a concentration of 4.2 × 10^6^ CFU/ml (~2 × 10^9^ cfu/kg metabolic wt/day). The required quantity of feed supplemented with fresh probiotics was fed twice a day. Piglets in groups A and B received intraperitoneal (ip) injections of 2 mg soluble ovalbumin (OVA) from chicken egg white (Sigma, Dorset UK) (systemic exposure) and 2 mg Quil-A adjuvant (Brenntag Biosector A/S, Frederikssund, Denmark) in 2 ml Phosphate Buffered Saline (PBS, Sigma) at 3 weeks of age to investigate the immune response against systemically administered novel protein. All piglets received the same immunization with ovalbumin (2 mg plus 2 mg Quil-A i.p.) at 9 weeks: for groups A and B this was their secondary response to ovalbumin; for groups C and D this was the primary response to ovalbumin. Piglets were bled by venepuncture at 3, 4, 5, and 9 weeks for collection of serum. Piglets were sedated with azaperone and euthanized with an overdose of barbiturate (Euthesate, Willows Francis Veterinary Ltd., Crawley, UK). At post-mortem, heart blood was recovered from all pigs.

**Table 1:** Diet composition of soya-based weaner feed (Calculated).

**Ingredient (%)**	**Soya-based weaner feed**
Whole dried egg	–
Extruded Full fat soya (unmodified, 35% protein, 19% fat)	17.6
High protein soya (48% protein, 2.7% fat)	12.2
Cooked wheat (MASHM)	19.4
Presco maize	19.7
Cooked naked oats	9.2
Dairy crest tint whey	8.8
Denatured skimmed milk -A	6.7
Dextrose	1.6
Vitamin and mineral mix	1.0
Dicalcium phosphate	1.5
Limestone Trical 130	0.5
L. lysine	0.4
L. threnine	0.1
Salt	0.1
Protein	21.2
Oil	11.2
Fiber	2.3
Ash	5.3
Moisture	9.9
NFE	50.1

***Vitamin and mineral mix** (Calculated units in finished feed)*.

*Vitamin A 16,000 iu/kg; vitamin D_3_ 2,000 iu/kg; vitamin E 250 iu/kg; vitamin K (menadione) 4 mg/kg; vitamin B_1_ 10 mg/kg; vitamin B_2_ 16 mg/kg; vitamin B_6_ 10 mg/kg; vitamin B12 0.05 mg/kg; Nicotinic acid 50 mg/kg; Pantothenic acid 30 mg/kg; Biotin (Vitamin K) 0.2 mg/kg; Vitamin C 200 mg/kg; Folic acid 3 mg/kg; Choline Chloride 300 mg/kg. **Trace minerals**: Copper 155 mg/kg; Iron 375 mg/kg; Zinc 110 mg/kg, Manganese 100 mg/kg; Cobalt 0.5 mg/kg; Iodine 1.2 mg/kg; Selenium 0.3 mg/kg*.

In experiment 2, 30 piglets were taken to the department of Clinical Veterinary Science at the University of Bristol at 12 h old (after colostrum intake) and housed in temperature controlled (28°C), slatted floored accommodation and supplied with heat lamps and vetbed® bedding. Piglets were housed together for 1 day whilst they learnt to drink bovine-based formula milk (Volac Ltd., Sleaford, Lincolnshire, UK). The nutritional composition of this formula is summarized in Table 2 and is similar to that given to human infants. In order to minimize the time taken for all of the piglets to learn to drink from bowls, piglets were housed in groups to learn by emulation. At two days old, the piglets were moved to individual units which also had slatted floors, heat lamps, and vetbed®, and were fed hourly for the duration of the trial (4 weeks). Once individually housed, the piglets were allocated into gender-balanced treatment groups which received either inulin (*n* = 10), starch (*n* = 10) supplementation in the formula, or received no supplementation (control group, *n* = 10) with 5 males and 5 females/group (Figure 1D). The commercially available product of chicory inulin, with a dry matter of 96.3%, containing >90% inulin with an average polymerization degree of 10%, a free sugar content of <10% and average chain length of 8–13 monomers (Sensu Ltd., Roosendaal, The Netherlands) or soluble starch from potato (Sigma) was added to the formula milk at a concentration of 0.45 g/Lt to give average inulin or starch consumption of 0.2 g/kg Metabolic Wt/day. Piglets were euthanized with an overdose of barbiturate (Euthesate, Willows Francis Veterinary Ltd., Crawley, UK) at 4 weeks old and tissues (mesenteric lymph node, distal jejunum, caecum and colon) were collected at post-mortem.

**Table 2:** Diet Composition of bovine-based formula milk (Calculated).

**Ingredient (%)**	**Formula (%)**
Spray dried instant whey powder	20.0
Whey protein concentrate (35%)	10.0
Whey Protein concentrate (80%)	4.0
Dairy crest tint whey	20.0
Denatured skimmed milk	43.4
Dextrose	0.8
Formula milk supplement^*^	1.0
Calcium formate	0.8
Protein	24.0
Oil	18.0
Fiber	7.5
Ash	3.5
Nitrogen free Extract NFE	47.0

* **Formula milk supplement** (units in finished feed).

*Vitamin A 16 mg/kg; vitamin D_3_ 2 mg/kg; vitamin E 250 mg/kg; vitamin K (menadione) 4 mg/kg; vitamin C 150 mg/kg; plus full complement of B group vitamins*.

### Tissue Culture

At sacrifice, 4 cm^2^ samples of intestinal mucosa (distal jejunum, caecum tip, and descending colon) and 1 cm^3^ of MLN (draining the distal jejunum) were collected and placed in cold sterile medium. Organ fragment culture (OFC) was carried out as previously described (44). Briefly, the sample were vigorously washed three times in Ca^2+^ and Mg^2+^-free Dulbecco's PBS (Sigma) containing 0.5 mM EDTA (Sigma), 1 M HEPES (Invitrogen, Paisley, UK) and 50 μg/ml gentamycin (Gibco), followed by 3 further washes in Ca^2+^ and Mg^2+^-free Dulbecco's PBS containing 1% HEPES and 50 μg/ml gentamycin before being placed in Roswell Park Memorial Institute (RPMI) 1640 (Sigma) containing 10% fetal calf serum (FCS) (PAA, UK), 200 mM L-Glutamine (Invitrogen), 20 U/ml streptomycin/penicillin (Invitrogen), and 50 μg/ml gentamycin (complete medium). All intestinal tissues were cut into fragments approximately 3 mm square while MLN was cut into 2 mm cubes and one fragment of tissue was placed in each of 6 individual wells of a 24 well culture plate (Corning Incorporated, UK) containing 1 ml of complete medium. Cultures were incubated at 37°C, 5% CO_2_, 100% humidity for 96 h, after which they were frozen at −20°C. The plates were defrosted and the spent medium from each of the 6 duplicate wells for each sample was pooled and refrozen for analysis of immunoglobulin content.

### Immunoglobulin Assays

Catching ELISA was carried out to determine total IgA and IgM in spent medium from OFC. Briefly, 96 well microplates were coated with either affinity purified goat anti-pig IgA or goat anti-pig IgM (Bethyl Laboratories, Montgomery, Texas, USA). Serial dilutions of serum samples and reference standard were added to coated plates and incubated for 2 h at room temperature. Bound immunoglobulins were detected using isotype-specific monoclonal antibodies (anti-pig IgA K61.1B4, anti-pig IgM K52.1C3 from Biorad) followed by horseradish peroxidase (HRP)-conjugated goat-anti-mouse IgG_1_. Concentrations of immunoglobulin subclasses were determined by interpolation of samples onto the reference standards.

### Antigen-Specific Immunoglobulin Assays

Serum samples were analyzed for anti-ovalbumin IgG_1_ antibodies 14 days after ip injection at 3 weeks (primary) and 9 weeks (secondary) by ELISA as previously described in detail (45). Briefly, 96 well microplates were coated with ovalbumin from chicken egg white (Sigma) before non-specific binding sites were blocked with 2% bovine serum albumin (BSA) (Sigma) in PBS-tween 20. After washing, serial dilutions of serum samples and reference standard were added to the plates. Reference standard was porcine serum obtained following hyperimmunization with ovalbumin. Bound anti-soya IgG_1_ antibodies were detected using isotype-specific monoclonal antibodies (clone K139 3C8, Biorad) followed by HRP-conjugated goat anti-mouse as above, and relative concentrations of antibody were determined by interpolation of samples onto the reference standards.

In order to compare changes in serum antibody generated by weaning and by injection of novel proteins in outbred animals, in which the starting levels differ, results are expressed as the ratio of antibody after manipulation to that before manipulation (the –fold change in antibody following exposure to OVA).

### Fluorescence Immunohistology

Proximal jejunum (avoiding Peyer's patches) was identified in each piglet at 75% along the length of the small intestine. The tissues were snap frozen then serial, 5 μm sections were cut using a Model OTF cryotome (Brights Instrument Company Ltd., Huntingdon. UK). Sections were air dried for 24 h then fixed by immersion in acetone for 15 min. Slides were dried prior to storage at −80°C.

Non-specific binding sites were blocked using 10% goat (serotec) and pig serum (Langford commercial abattoir) and the samples were then stained with monoclonal antibodies. The following monoclonal antibodies were used: anti-porcine CD4 (clone MIL17, BioRad); anti-porcine CD172/Sirp-α (clone 74-22-15 Cambridge Bioscience), anti-porcine intestinal capillary endothelium (clone MIL11, Bio-Rad), anti-porcine MHC class II DR (clone MSA-3, BioRad), Rat anti-mouse Foxp3 (clone FJK-16s, ThermoFisher), anti-porcine CD25 (clone K231.3B2, BioRad), rabbit anti-mouse Zona Occludin-1 (ZO-1, clone 61-7300, BioRad), anti-porcine CD45 (clone k252-1e4, BioRad), and mouse anti-rat E-cadherin (clone DECMA-1, AbCam). Binding was detected with the following: goat anti-mouse IgG_2b_ TRITC (Southern Biotechnology), goat anti-mouse IgG_1_ FITC (Southern Biotechnology), biotinylated goat anti-mouse IgE (Southern Biotechnology) detected with AMCA-Avidin D (Vector Laboratories), goat anti-mouse IgG_2a_ AlexaFluor 633 (Invitrogen), goat anti-rat IgG FITC (Stratech), biotinylated goat anti-mouse IgG_1_ (Southern Biotechnology), detected with AMCA-Avidin D (Vector Laboratories), goat anti-rabbit TRITC (Southern Biotechnology), and goat anti-mouse IgG_1_ TRITC (Southern Biotechnology). Non-specific binding was prevented by 5% pig serum, 5% goat serum, and 5% rat serum in PBS. Slides were mounted using Vectashield (Vector Laboratories). Negative control slides were prepared in conjunction with each positive slide.

### Image Capture and Analysis

Image capture and proportional area of CD4, CD172, MIL11, MHCII, ZO-1, CD45, and E-cadherin positive staining were analyzed using an in-house macro and ImageJ version 1.44 (46). Briefly, ten, 16 bit grayscale images were captured for each piglet along the small intestine resulting in either 70 (Figure 1A) or 50 (Figure 1D) representational images for each sex/treatment group, using a Leica DMR-B fluorescence microscope fitted with appropriate fluorescence filters. The proportion of positive pixels in each color channel was measured using a specifically developed in-house macro. This allowed quantification of positive staining by the primary antibodies and values were logged to achieve normal distributions, as verified by Inman et al. (46). Because expression of Foxp3 is nuclear, numbers of CD4^+^CD25^+^Foxp3^+^ were analyzed manually.

### Statistical Analysis

Multivariable linear regression was carried out using IBM SPSS statistics (IBM, Chicago, IL, USA) on the quantitative immunofluorescence and on antibody and immunoglobulin data using piglet as the experimental unit and age, antigenic challenge (ovalbumin i.p.), litter, sex, and nutritional supplementation (probiotic, prebiotic, or starch) as variables where appropriate. In order to avoid overfitting, simplified models where used in which the only interactions considered were between sex and probiotic, and sex, probiotic, and immunization in experiment 1 or sex, tissue, and prebiotic or starch in experiment 2. Individual differences between treatment groups and sex were determined by least-significant differences as in our previous experiments (47). The proportion of pixels positive for CD4, CD172, MIL11, MHCIIDR, ZO-1, CD45, E-cadherin, and the number of CD4^+^CD25^+^Foxp3^+^ cells.mm^2^ were analyzed as individual, dependant variables. Where multiple dependant variables were analyzed, Bonferroni corrections were used to avoid type 1 errors.

## Results

### Sex Determines Levels of Antibody to Ovalbumin After Weaning

**Figure 2:**
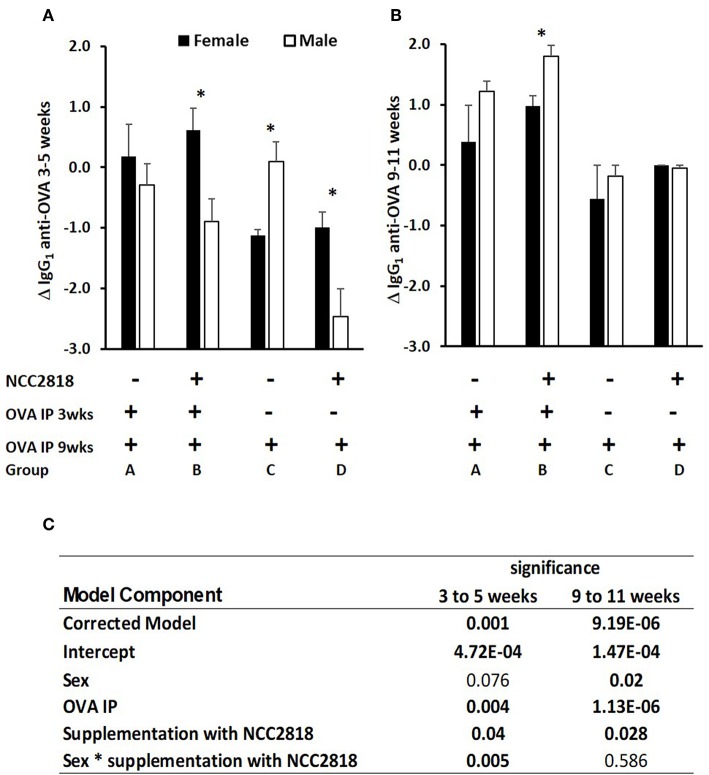
Difference between systemic ovalbumin (OVA)-specific IgG_1_ antibody levels from 3 to 5 weeks **(A)** and 9–11 weeks **(B)** of age in all soya-fed piglets. Increase in OVA-specific IgG_1_ antibody in response to priming and recall with intraperitoneal (IP) OVA with QuilA adjuvant in male (white bars, *p* < 0.001), but not in female (black bars) piglets weaned onto a soya protein-based diet. Significant interactions are presented in **(C)**. Samples were obtained 14 days after both IP OVA exposures. In order to compare changes in serum antibody generated by weaning and by injection of novel proteins in outbred animals, in which the starting levels differ, results are expressed as the ratio of antibody after manipulation to that before manipulation (the Log_10_-fold change in antibody following exposure to OVA). *significant difference between males and females within the experimental group *p* < 0.05; Error bars = SEM.

In experiment 1, antibody to ovalbumin was detected in all groups of animals at 3 weeks old, reflecting a combination of response to immunization, maternally-derived antibody and background crossreactive antibody. Between 3 and 5 weeks old, levels in unimmunized piglets generally declined, reflecting the decline in maternally-derived antibody. In piglets immunized at 3 weeks old, the decline in levels of antibody to 5 weeks was, generally, reduced by comparison with unimmunized piglets reflecting active immune responses to immunization against this background of decreasing maternal antibody (Figure 2A). However, although statistical analysis confirmed significant effects of immunization (*p* = 0.001), it also indicated significant effects of sex and of probiotic. Importantly, there was a significant two-way interaction between sex and probiotic (*p* = 0.002) and an additional but less significant three-way interaction between sex, probiotic, and immunization (*p* = 0.025), indicating that sex differences were present both in the immunized and unimmunized groups but were stronger on the unimmunized (Figure 2C).

In piglets immunized at 9 weeks old, the effect of immunization was more marked (*p* = 0.000002, Figure 2B), likely because of the absence of maternally derived antibody, and because all piglets received an immunization, the difference being whether it was their first or second immunization. Sex and administration of probiotic also had significant effects, females making less antibody and probiotic supplemented making more. Unlike at 3–5 weeks, there were no significant interactions.

### Immunoglobulin Production in Response to Dietary Inulin and Starch Differed Significantly Between the Sexes in Both Lymphoid and Non-lymphoid Tissues

Total IgA and IgM were quantified in organ fragment culture medium from all piglets in experiment 2 (controls, inulin and starch supplemented). There were highly significant differences between tissues in the amounts of IgA and IgM produced irrespective of treatment (*p* < 0.0001, *p* = 0.0020, respectively). There was no significant effect of inulin or starch supplementation on IgA production when the results were analyzed without taking sex into account. However, there were also overall significant differences in response to treatment in IgM production in all tissues (*p* = 0.002): both inulin (1.76 ± 0.16 log_10_ μg/ml) and starch (2.04 ± 0.19 log_10_ μg/ml) resulted in increased IgM production compared to the control piglets (1.29 ± 0.09 log_10_ μg/ml) when the results were analyzed without taking sex into account.

**Figure 3:**
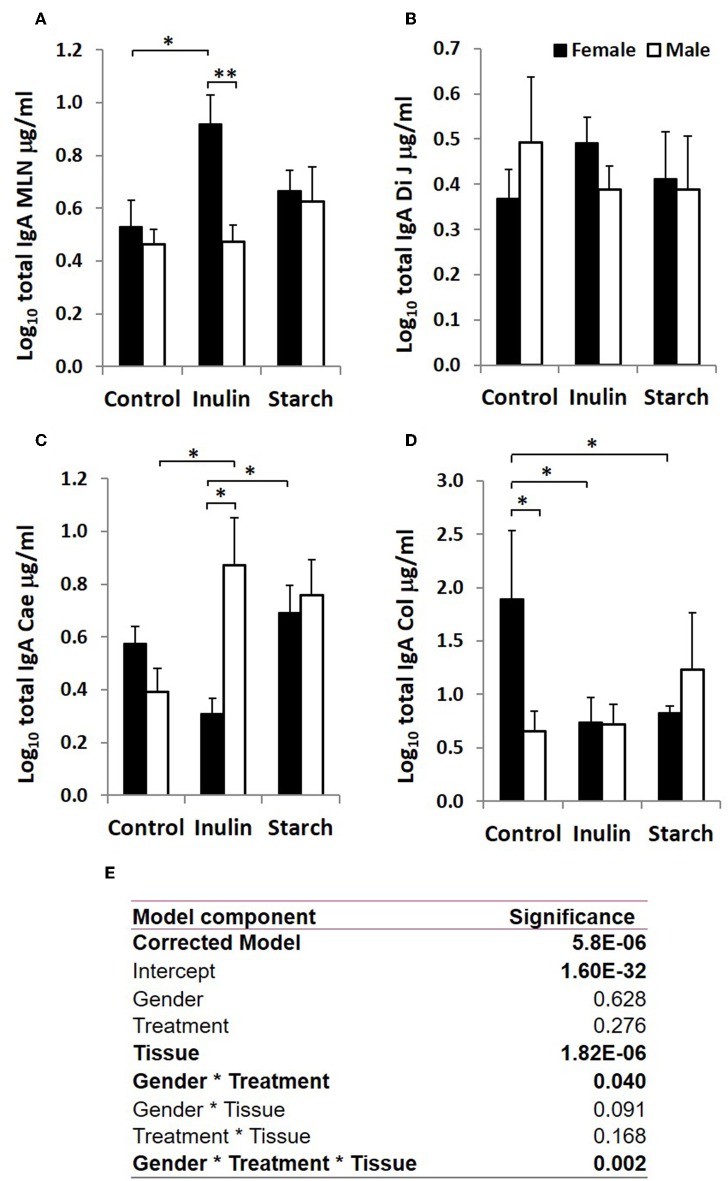
Total IgA production (μg/ml, log-transformed) by organ fragment cultures from organized lymphoid tissue (mesenteric lymph node, MLN) **(A)** and non-lymphoid tissues; distal jejunum **(B)**, caecum **(C)**, and colon **(D)** from female (black bars) and male (white bars) piglets supplemented with inulin or starch, and non-supplemented (control) groups. Six replicates were pooled to generate each sample for analysis. Table of significances **(E)**. *p < 0.05; **p < 0.01. Error bars SEM (*n* = 5/sex/treatment group).

**Figure 4:**
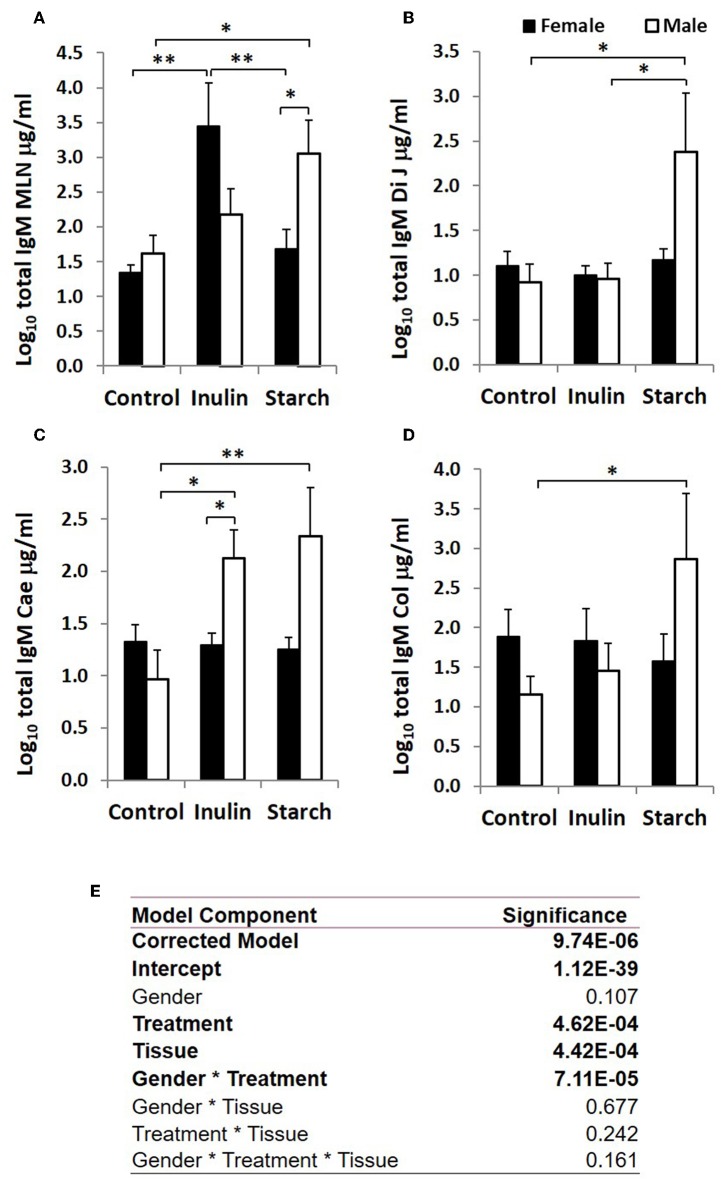
Total IgM production (μg/ml, log-transformed) by organ fragment cultures (OFC) from organized lymphoid tissue (mesenteric lymph node, MLN) **(A)** and non-lymphoid tissues; distal jejunum **(B)**, caecum **(C)**, and colon **(D)** from female (black bars) and male (white bars) piglets supplemented with inulin or starch, and non-supplemented (control) groups. Six replicates were pooled to generate each sample for analysis. Table of significances **(E)**. *p < 0.05; **p < 0.01. Error bars SEM (*n* = 5/sex/treatment group).

When sex was included as a factor in the models, there were statistically significant interactions between sex, tissue and treatment (*p* = 0.002, Figure 3) in IgA production, and between sex and treatment (*p* < 0.001, Figure 4) in IgM production. In females, the increased IgA and IgM synthesis in response to inulin supplementation was apparent in the organized lymphoid tissues of the MLN whereas in males, it was apparent in the primary fermentation chamber, the caecum. Starch supplementation had no effect on females but increased IgM synthesis in all tissues in males.

### The Development of Immune-Associated Cells in the Intestinal Mucosa Is Dependent on Sex, and in Response to Dietary Supplementation With Inulin and Starch

**Figure 5:**
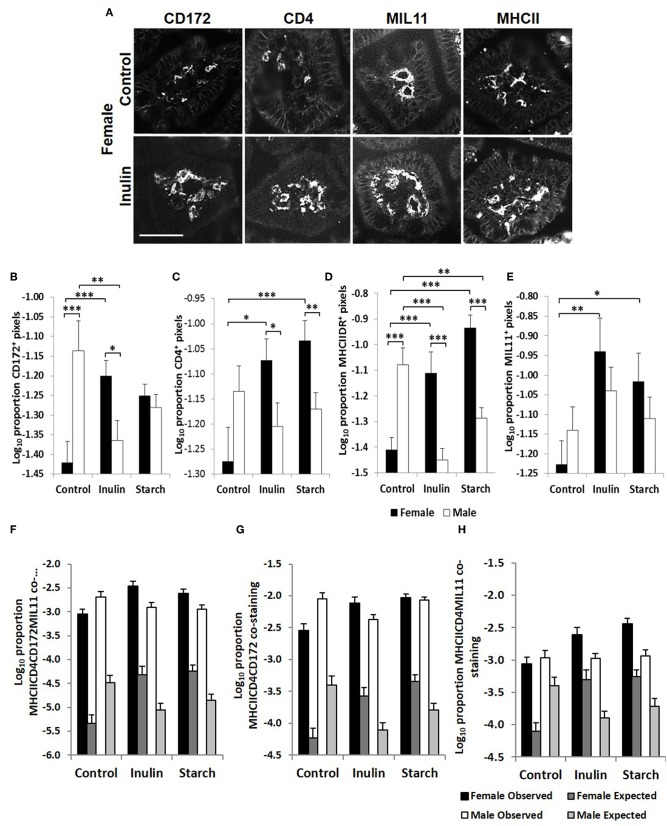
Representation of fluorescence immunohistology from the distal jejunum *lamina propria* of a 28 day old female control (no dietary supplementation) and inulin supplemented piglets showing CD172 (Sirpα), CD4, and MIL11 (capillary endothelium) MHCIIDR positive staining **(A)** Bar = 100 μm. Quantitative analysis of proportion positive pixels by fluorescence immunohistology (log transformed) of CD172 **(B)**, CD4 **(C)**, MHCIIDR **(D)**, and MIL11 **(E)** positive staining in the distal jejunum *lamina propria* of 28 day old piglets by treatment (unsupplemented control, inulin, or starch supplemented) and by sex (females = black bars, males = white bars). Observed (dark gray = females, light gray = males) area of the indicated stains compared with the expected (black = female, white = male) area (i.e., the area of staining predicted from a null hypothesis that each element of positive staining is distributed randomly and independently) for CD4CD172MHCIIMIL11 **(F)**, CD4CD172MHCII **(G)**, and CD4MIL11MHCII **(H)** co-staining. Ten individual images were analyzed for each sample, *p < 0.05; **p < 0.01; ***p < 0.001. Error bars SEM (*n* = 5/sex/treatment group).

Fluorescence immunohistology was used to assess sex differences in the development of the immune system by quantifying expression of immune-associated proteins in the intestinal mucosa of the distal jejunum in control piglets, and in response to dietary supplementation with inulin or starch at 28 days old. Representative images are shown in Figure 5A. Supplementation with inulin resulted in a significant increase in the proportion of area (log transformed) staining positive for immune-associated proteins (dendritic cell surface marker CD172/Sirp-α, *p* < 0.001, Figure 5B; CD4, *p* < 0.05, Figure 5C; MHCIIDR, *p* < 0.001, Figure 5D; MIL11 capillary endothelium, *p* < 0.01, Figure 5E) in females (compared to female controls), whereas in males, inulin supplementation resulted in a significant decrease in the area staining positive for CD172/Sirp-α (*p* < 0.01) and MHCIIDR (*p* < 0.001) compared to control males.

Supplementation with starch was also linked to increases in the proportion of positive staining for CD4 (*p* < 0.001), MIL11 (*p* < 0.05), and MHCIIDR (*p* < 0.001) in females compared to their control counterparts, but not in males. As with supplementation with inulin, intervention with dietary starch resulted in significant decreases in expression of MHCII (*p* < 0.01) in males compared to unsupplemented male controls. The proportion of positive staining for CD172/Sirp-α and MHCIIDR was significantly higher in males compared to females in the control groups (*p* < 0.001).

**Figure 6:**
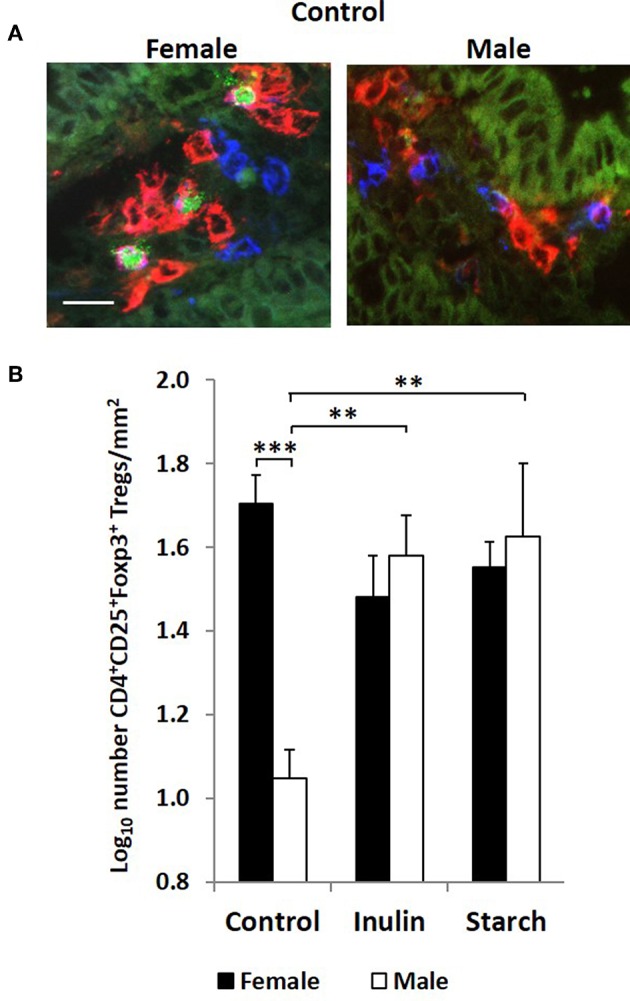
Representation of 3 color fluorescence immunohistology of CD4 (red), CD25 (blue), Foxp3 (green), and CD4CD25 (magenta) positive staining in the distal jejunum *lamina propria* of a 28 day old female and male control (no dietary supplementation) piglets **(A)**. Bar = 10 μm. Quantitative analysis (log transformed) of CD4^+^CD25^+^Foxp3^+^ cells/mm^2^ of distal jejunum *lamina propria* in 28 day old piglets by treatment (unsupplemented control, inulin or starch supplemented) and by sex (females = black bars, males = white bars) **(B)**. Ten individual images were analyzed for each sample. *p < 0.05; **p < 0.01; ***p < 0.001. Error bars SEM (*n* = 5/sex/treatment group).

Interestingly, although control males expressed higher levels of CD4^+^ staining in the intestinal mucosa of the distal jejunum compared to their female counterparts, they had significantly fewer CD4^+^CD25^+^Foxp3^+^ cells/mm^2^ (representative images in Figure 6A) in this tissue than the female control piglets (*p* < 0.001, Figure 6B). Supplementation with both inulin and starch resulted in an increase in the number of CD4^+^CD25^+^Foxp3^+^ cells/mm^2^ (*p* < 0.01 in both cases) in males. However, neither dietary supplement resulted in significant changes in the number of cells with this phenotype in female piglets.

In previous studies, we have compared the observed overlap between CD4, MHCII, CD172, and MIL11 to that expected if the distribution was random and independent, to show that CD4^+^ T-cells in the intestinal *lamina propria* do interact directly both with dendritic cells and with endothelial cells *in vivo* and demonstrated that both cell types are capable of antigen presentation (48). Sex-related differences in recruitment of CD172, CD4, and MHCII^+^ cells could be resulting in functional differences in the number of interactions between these cell types or the recruited cells could be simply “in transit,” not engaging in any kind of surveillance. In all cases, the observed overlap was significantly greater than that expected (*p* < 0.000001). In the studies reported here, increases associated with inulin supplementation in females in total areas of CD4, CD172, MHCII, and MIL11 and differences between males and females remained significantly different from expected (Figures 5F–H), indicating that differences in the number of recruited cells are likely to contribute to quantitative changes in the local antigen presenting environment.

### Intestinal Barrier Function Is Increased by Supplementation With Both Inulin and Starch in a Sex Independent Manner

**Figure 7:**
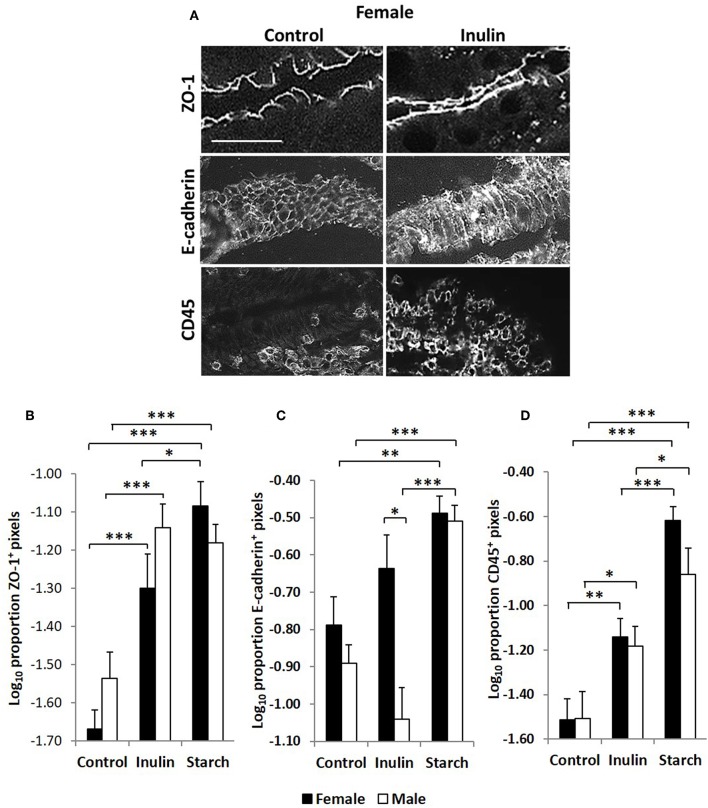
Representation of fluorescence immunohistology from the distal jejunum epithelium of a 28 day old female control (no dietary supplementation) and inulin supplemented piglets showing ZO-1 (zona occludin 1), E-cadherin and CD45 positive staining **(A)**. Bar = 100 μm. Quantitative analysis of proportion positive pixels by fluorescence immunohistology (log transformed) of ZO-1 **(B)**, E-cadherin **(C)**, and CD45 **(D)** positive staining in the distal jejunum epithelium of 28 day old piglets by treatment (unsupplemented control, inulin or starch supplemented) and by sex (females = black bars, males = white bars). Ten individual images were analyzed for each sample. *p < 0.05; **p < 0.01; ***p < 0.001. Error bars SEM (*n* = 5/sex/treatment group).

Quantitative fluorescence immunohistology was used to assess expression of proteins associated with barrier-function in the epithelium of the distal jejunum in response to supplementation with inulin and starch in 28 day old piglets. Representative images are shown in Figure 7A. Expression of the tight cell junction associated protein Zonaoccludin-1 (ZO-1) and the lymphocyte common antigen CD45 were increased in the epithelium in response to dietary supplementation with both inulin and starch and differences were not observed between female and male piglets (Figures 7B–D). In contrast, inulin supplementation resulted in significant differences between the sexes in expression of E-cadherin, females expressing higher levels than males (*p* < 0.05). Supplementation with starch was linked to increases in both sexes compared to their control counterparts (Figure 7C, *p* < 0.001).

### Antibody Responses to Novel Protein Fed at Weaning Are Higher in Females Than Males, and Is Increased by Supplementation With *Bifidobacterium lactis* NCC2818 in Both Sexes

**Figure 8:**
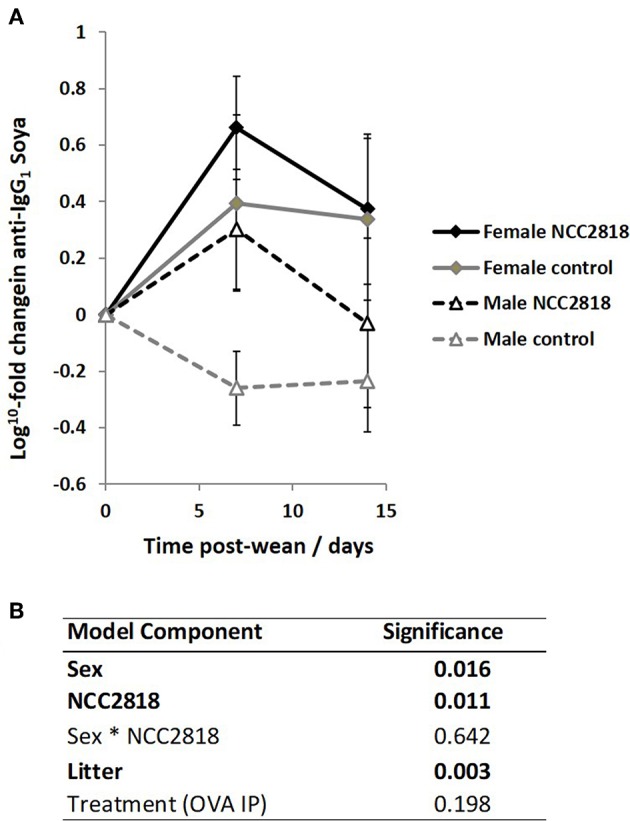
Increased systemic soya-specific IgG_1_ antibody in response to weaning onto a soya-based diet in females (solid lines), compared to males (dotted lines) in piglets at 7 days post wean (*p* = 0.016). Supplementation with *B. lactis* NCC2818 (black lines) also resulted in increased systemic soya-specific IgG_1_ antibody production compared to unsupplemented piglets (gray lines) in males and females (*p* = 0.011). Figures represent aggregated data from groups A and C (Not supplemented with NCC2818) and groups B and D (supplemented with NCC2818). A dilution series was analyzed for each sample **(A)**. Table of significances **(B)**. Error bars SEM; (*n* = 7/sex/treatment group).

As we have previously reported (44), there was an increase in IgG_1_ anti-soya antibody in serum of piglets after weaning at 3 weeks old onto soya-based diets. Statistical analysis using sex, NCC2818 supplementation, litter, and treatment as fixed factors demonstrated that there was no significant effect of ovalbumin injection on the level of anti-soya antibody produced in response to weaning onto soya protein (Figure 8B, *p* = 0.198): therefore, for display (Figure 8A), animals in groups A and C (shown in Figure 1) were grouped together as were animals in groups B and D. The level of antibody produced after weaning varied between litters, but was significantly increased by supplementation with NCC2818 (Figure 8, *p* = 0.0111). However, there were also significant differences between soya-fed males and females in the level of response: 7 days after initial exposure, both unsupplemented and supplemented female piglets made significantly more IgG_1_ anti-soya antibody than their male counterparts (*p* = 0.016). No interaction between sex and probiotic supplementation was observed at this time point (*p* = 0.642). In addition, no significant differences were observed between the sexes by 14 days after weaning, or in response to supplementation with *B. lactis* NCC2818. There were no significant sex differences in IgG_2_ anti-soya antibody response to novel soya protein at weaning (data not shown).

## Discussion

Sex disparity in immune responses is well-documented. However, sex differences in immune development during early infancy are still poorly understood, despite their clear importance for the development of stratified healthcare in both human and veterinary medicine. Here, using an outbred piglet model, we have identified several important differences in early immune development between the sexes as early as 28 days of age. These include CD172^+^ (Sirp-α) antigen presenting numbers and expression of MHCIIDR in the intestinal mucosa, regulatory T-cell populations, mucosal IgA production and systemic antibody response to injected novel ovalbumin.

Decreased intestinal barrier function is emerging as an early predisposing factor for metabolic and immune dysfunction that underlies several chronic degenerative diseases in later life (49). In healthy adult males, intestinal barrier function is generally less effective than in females and less sensitive to NSAID-induced perturbation. Barrier integrity is also more variable in males compared to females and, in some cases, can decrease to levels similar to those observed in inflammatory bowel conditions (50). Despite this, our study did not find significant sexual disparity in expression of epithelial ZO-1, a tight cell junction (TCJ) protein associated with barrier function (51, 52), or in E-cadherin which has an important role in maintaining barrier integrity under homeostatic condition (53) in 28 day old piglets. This suggests that the differential development of gut barrier function may occur later in life than infancy and could be linked to differences in sex hormones which occur from puberty onwards (54, 55).

However, despite the observed similarities in barrier function between male and female piglets and, presumably, in levels of exposure to antigen crossing this barrier, there was sex disparity in both expression of antigen-presenting cell (APC) and of CD4^+^ T-cells -associated proteins in the intestinal mucosa. CD172^+^ (Sirp-α) APC and MHCIIDR expression were both significantly higher in control, unsupplemented males than females. Sexual bias in MHC I-associated CD8^+^ cell expansion in adults with multiple sclerosis has previously been reported (56) but sex differences in antigen presentation in healthy young animals has not. In our study, control females presented with significantly less CD172^+^ APC and less (though not significant) CD4^+^ staining in the submucosa of the distal jejunum at 28 days compared to unsupplemented males. Within the CD4^+^ T-cell population in this location, females had significantly more regulatory T-lymphocytes (Tregs) than males. Together, the lack of APC and CD4^+^ staining and the higher number of Foxp3^+^ T-cells suggests that females had reduced potential to generate mucosal immune responses to antigens translocating into the *lamina propria* from the lumen. While this is not consistent with reports in human adults, where autoimmune diseases and allergies generally have higher prevalence in females compared to males (1, 4, 5) the opposite has been reported in children under 18 years old where 64.35% of a food allergy cohort were male and 35.65% were female (57). In addition, in a longitudinal study from birth to late adulthood, asthma, allergic rhinitis, and eczema all exhibited male predominance in childhood that reversed during adolescence (58). This aligns with our results where young males presented with a less regulated gut mucosal microenvironment, whereas females were potentially better able to regulate mucosal immune responses.

However, although control female piglets appeared to have increased potential for local immune regulation in the gut mucosa compared to their male counterparts, they produced significantly increased systemic IgG antibody in response to novel injected and oral antigens than control males around weaning at 4 weeks old, suggesting greater ability to mount systemic immune responses at this age. Although it is difficult to make accurate analogies between piglet and human infant age and stage of development, we do also demonstrate a switch to males producing increased systemic antibody to injected OVA with exposure at 9 weeks old. Thus, our results support a model in which normal, healthy females regulate mucosal responses better than males in early neonatal life but mount stronger systemic responses, but this switches around as the animals age.

In addition to sex effects in unsupplemented piglets, the studies reported here demonstrated differences between males and females supplemented with the prebiotic inulin. Consistent with other studies which show that supplementation with inulin resulted in significant increases in gene transcripts associated with intestinal barrier function (59), we show that inulin was associated with increased TCJ protein expression in the epithelium of the distal jejunum at 28 days. However, the effects of inulin on barrier function-related proteins were not different between sexes. Our data are consistent with previous evidence suggesting that younger females have greater control over local, mucosal responses to antigen compared to males, inulin supplementation resulted in significantly increased IgA and IgM production by caecal mucosa in males but not in females, whereas mesenteric lymph node tissues from supplemented females produced more than from males. Interestingly, both IgA and IgM production by the MLN was higher in females than males following supplementation. This may indicate that plasma cells are more likely to differentiate and be retained in the MLN in females but more likely to recirculate to the intestinal mucosa in males.

How antigen is presented to the immune system is clearly an important determinant in driving immune development in young mammals. We assessed only the mucosal CD172^+^ APC population and there could also be sex disparity in ratios of other APC subsets (those defined, for example, by combinations of CD172, CD16, and CD11R1 surface expression) which ultimately result in differences in early T-cell differentiation between the sexes. We have previously demonstrated that early exposure of piglets to farm environments significantly affects antigen presentation by both APC and stromal cell subsets in the intestinal mucosa (60). It is well-established that early exposure of human infants to farm environments is linked with a reduced risk of allergy (i.e., the hygiene hypothesis). One possibility is that some component within early farm exposure may differentially affect male and female infants, generating a more regulatory mucosal microenvironment similar to that which occurs naturally in females. Our previous studies clearly suggest that early environment does affect mucosal regulatory environments, and specific components of a human microbiome have been correlated with numbers of regulatory T-cells (61). However, since this latter was a germ-free pig model, it does not preclude the possibility that factors other than the microbiome are also likely to be involved. It would be interesting to explore whether there is sex disparity in the protective effect of farm environments and other early-life factors: to the best of our knowledge, this is yet to be assessed.

Despite limited understanding of the mechanisms of action of both prebiotics and probiotics, their use in functional foods and in clinical applications has increased rapidly over recent years. Meta-analyses of randomized controlled trials using probiotics in infants show promising results in the prevention and treatment of common diseases such as diarrhea and allergies (62–65). In contrast, other studies have not shown that probiotic supplementation in healthy infants has any discernible health benefits (66). In such trials, although gestational age and weight at birth, attendance at pre-school facilities, the presence of siblings and pet ownership are often taken into account during analyses, sex seldom is. However, we have previously shown that diet also influences the response to probiotics (44) and the studies here further demonstrate that some of the impacts of both prebiotic and probiotics on immune development in young piglets were also sex-specific. The combination of environmental, genetic, and phenotypic influences on the response to functional foods may, in part, begin to explain inconsistencies in the outcomes of many reported studies. This also raises the possibility that the beneficial effects of certain prebiotics and probiotics may differ between sexes, providing a potential target for stratified interventions, and is currently an avenue which remains unexplored. As an example, this may be particularly important in the application of prophylactic probiotic therapy in premature babies with increased risk of necrotizing enterocolitis and late-onset sepsis (67).

In conclusion, we present evidence that important immunological differences occur between healthy female and male outbred piglets, even as early as 28 days old. This suggests that there will also be differences in predisposition to immunological diseases in infants, as occurs in adults, but that infant sex biases may be qualitatively different from those which occur in adults. Similarly, we show that some of the effects of probiotics and prebiotics on developing immune systems appear to be different between the sexes. This is, perhaps, unsurprising considering the underlying immunological differences we observed. However, it does raise the possibility that prebiotics and probiotics may need to be targeted more specifically across age and sex in order to achieve optimum health benefits. Outbred pigs, where direct, controlled interventions in neonates can be followed by extensive, invasive tissue collection and analysis, may present an appropriate model for studies of the mechanisms underlying sex disparity in immune development and consequent sex-specific responses to functional foods.

## Data Availability Statement

The datasets generated for this study are available on request to the corresponding author.

## Ethics Statement

Animal housing and experimental procedures were all performed at the University of Bristol Veterinary Science School in accordance with local ethical guidelines. All experiments were reviewed and approved by the Bristol Animal Welfare and Ethical Review Body (AWERB) and were performed under a UK Home Office License.

## Author Contributions

ML, MM, MB, GW, MA, and CP conceived the idea. ML, MB, and ZC performed the experiments. ML collected and analyzed the data, performed statistical analyses, wrote the manuscript, and responsible for the overall direction of the paper. ML, MB, GW, MA, and CP interpreted the data. ZC provided the T_reg_ data. ML and MB provided expertise on the pig model and pig immunology. All authors reviewed the manuscript and approved the version to be published.

### Conflict of Interest

The authors declare that the research was conducted in the absence of any commercial or financial relationships that could be construed as a potential conflict of interest.
